# Inoculation of Soil with Plant Growth Promoting Bacteria Producing 1-Aminocyclopropane-1-Carboxylate Deaminase or Expression of the Corresponding *acdS* Gene in Transgenic Plants Increases Salinity Tolerance in *Camelina sativa*

**DOI:** 10.3389/fmicb.2016.01966

**Published:** 2016-12-16

**Authors:** Zohreh Heydarian, Min Yu, Margaret Gruber, Bernard R. Glick, Rong Zhou, Dwayne D. Hegedus

**Affiliations:** ^1^Agriculture and Agri-Food Canada, SaskatoonSK, Canada; ^2^Department of Biotechnology, School of Agriculture, Shiraz UniversityShiraz, Iran; ^3^Department of Biology, University of Waterloo, WaterlooON, Canada; ^4^Department of Food and Bioproduct Sciences, University of Saskatchewan, SaskatoonSK, Canada

**Keywords:** *Camelina sativa*, salinity tolerance, 1-aminocyclopropane-1-carboxylate deaminase, plant growth promoting bacteria, transgenic plants

## Abstract

*Camelina sativa* (camelina) is an oilseed crop touted for use on marginal lands; however, it is no more tolerant of soil salinity than traditional crops, such as canola. Plant growth-promoting bacteria (PGPB) that produce 1-aminocyclopropane-1-carboxylate deaminase (ACC deaminase) facilitate plant growth in the presence of abiotic stresses by reducing stress ethylene. Rhizospheric and endophytic PGPB and the corresponding *acdS-* mutants of the latter were examined for their ability to enhance tolerance to salt in camelina. Stimulation of growth and tolerance to salt was correlated with ACC deaminase production. Inoculation of soil with wild-type PGPB led to increased shoot length in the absence of salt, and increased seed production by approximately 30–50% under moderately saline conditions. The effect of ACC deaminase was further examined in transgenic camelina expressing a bacterial gene encoding ACC deaminase (*acdS*) under the regulation of the CaMV *35S* promoter or the root-specific *rolD* promoter. Lines expressing *acdS*, in particular those using the *rolD* promoter, showed less decline in root length and weight, increased seed production, better seed quality and higher levels of seed oil production under salt stress. This study clearly demonstrates the potential benefit of using either PGPB that produce ACC deaminase or transgenic plants expressing the *acdS* gene under the control of a root-specific promoter to facilitate plant growth, seed production and seed quality on land that is not normally suitable for the majority of crops due to high salt content.

## Introduction

Salinity stress is a worldwide agricultural problem that negatively affects plant growth and production. Across the Canadian Prairies, 20 million hectares alone are seriously affected by salinity caused by high concentration of salts dissolved in soil water (Steppuhn, 2013). Furthermore, global warming is expected to increase soil salinity due to higher evaporation and lower precipitation. Irrigation from both ground and surface water sources increases soil salinity over time as dissolved minerals accumulate in the soil. Collectively, salinity issues affect 20% of cultivated land and up to 50% of irrigated land worldwide (Cheng et al., 2012).

*Camelina sativa* (camelina), a member of the Brassicaceae family and a close relative of the model plant *Arabidopsis thaliana* and the oilseed *Brassica* crops, such as canola and Indian mustard, was grown extensively in medieval Europe for food and fuel. Renewed interest in this ancient crop stems from demand for species that can diversify annual crop rotation portfolios, with a lower environmental footprint and with potential to produce high value secondary products. Camelina is currently being grown on limited acreage to provide oil feedstock for the biofuel and bio-lubricant sectors (Blackshaw et al., 2011; Li and Mupondwa, 2014). The high levels of polyunsaturated fatty acids, in particular α-linolenic acid (38% total fatty acid), also make it an attractive source of ω–3 fatty acids in food and feed (Hixson and Parrish, 2014; Hixson et al., 2014). Camelina is amenable to production practices used for current crops, such as canola and soybean. It can grow on marginal lands (Putnam et al., 1993), has enhanced drought and cold tolerance, displays early maturation and requires fewer inputs compared to other oilseeds (Vollmann et al., 1996; Zubr, 2003). It is also naturally resistant to diseases, such as blackspot (Sharma et al., 2002), blackleg (Li et al., 2005), and stem rot (Eynck et al., 2012), as well as insect pests, such as the flea beetle and diamondback moth (Deng et al., 2002; Henderson et al., 2004; Soroka et al., 2014), which aﬄict canola. As an industrial oilseed crop, camelina would ideally be grown on marginal lands that are not well-suited for food crop production. However, growth, seed yield and oil content studies showed that camelina does not perform as well as canola when grown under conditions that simulate prairie saline soils; in particular camelina seed yield is much lower under saline conditions (Steppuhn et al., 2010).

High concentrations of salt cause ion imbalance and hyperosmotic pressure, leading to oxidative stress. This manifests as changes in molecular and biochemical pathways involved in stress tolerance and ultimately in plant physiological adaptations (Parihar et al., 2015). Plant growth-promoting bacteria (PGPB) are soil bacteria that facilitate plant growth and are often found in association with plant roots (rhizospheric), and sometimes within plant tissues (endophytic) (Bacon and Hinton, 2006; Glick, 2015). PGPB promote plant growth and development through a variety of mechanisms including the synthesis of indole acetic acid (Duca et al., 2014), production of metal-accumulating siderophores (Farwell et al., 2007; Ali et al., 2014), improved nitrogen fixation (Compant et al., 2005), improved ammonia production (Marques et al., 2010) and solubilization of mineral phosphate (Verma et al., 2001). However, some PGPB also produce the enzyme 1-aminocyclopropane-1-carboxylate deaminase (ACC deaminase). This enzyme converts the ethylene precursor ACC to α-ketobutyrate and ammonia and promotes plant growth, especially during stress conditions, by reducing the level of stress ethylene to below the point where it is inhibitory to growth (Glick, 1995, 2012; Singh et al., 2015). Salinity stress has been shown to elevate ethylene levels (Mayak et al., 2004), which affects almost all aspects of plant growth and development, including the response to biotic and abiotic stresses (Cao et al., 2008). Tomato, canola, maize, groundnut, cotton, cucumber, and red pepper were more resistant to high salinity when treated with various PGPB strains producing ACC deaminase (Mayak et al., 2004; Nadeem et al., 2007; Saravanakumar and Samiyappan, 2007; Yue et al., 2007; Gamalero et al., 2010; Siddikee et al., 2011; Cheng et al., 2012; Gamalero and Glick, 2015).

In this study, we examined the effect of rhizospheric and endophytic plant growth-promoting Pseudomonads that produce ACC deaminase on the growth and development of camelina under saline conditions. We also examined the effect of expressing a bacterial gene encoding ACC deaminase (*acdS*) in transgenic camelina under the control of a broadly constitutive and a root-specific promoter.

## Materials and Methods

### Bacterial Strains

Several PGPB that produce ACC deaminase were tested for their ability to increase salinity tolerance in camelina. The PGPB strains included the rhizosphere-associated *Pseudomonas putida* UW4 (Duan et al., 2013) originally isolated from common reeds (Glick, 1995), and two root endophytes, *P. migulae* 8R6 and *P. fluoresces* YsS6 (Rashid et al., 2012). In addition, the effect of two *acdS* mutant endophytic strains, 8R6M and YsS6M, was examined. These were constructed previously by insertion of a transposon containing the tetracycline resistance gene at position 237 in the *acdS* gene of *P. migulae* 8R6 and at position 323 in the *acdS* gene of *P. fluorescens* YsS6. The *acdS* activity of the mutants was reported to be 0.11 and 0.03 μmol μg^-1^ h^-1^ for 8R6 and YsS6, respectively (Ali et al., 2014). The strains were verified by amplification of the *acdS* gene using primer set 1 (5′-ATGAACCTGAATCGTTTTGA-3′ and 5′-TCAGCCGTTGCGAAACAGGA-3′) for *P. putida* UW4 and *P. fluorescens* YsS6 and YsS6M or primer set 2 (5′-ACAGGAAGCTGTAGGCGTTC-3′ 5′-CTGTATGCCAAGCGTGAAGA-3′) for *P. migulae* 8R6 and 8R6M as per Ali et al. (2014).

### ACC Deaminase Vector Construction and Plant Transformation

The *acdS* gene was previously isolated from *P. putida* UW4 and inserted into plasmid pKYLX71.1 under the direction of either the double cauliflower mosaic virus (CaMV) *35S* promoter (pB171) or the *rolD* promoter from *Agrobacterium rhizogenes* (pB172) (Elmayan and Tepfer, 1995; Shah et al., 1998). The UW4 ACC deaminase is 99% identical (amino acid level) to that of YsS6 and 98% identical to 8R6. The cassette containing the *acdS* gene driven by the CaMV *35S* promoter was released by digestion with *Sma*I and *Xba*I from pB171 and ligated into the pORE-O3 binary vector digested with *Sfo*I and *Xba*I. pORE-O3 carries the kanamycin and phosphinothricin acetyltransferase selectable markers (Coutu et al., 2007). The *acdS* gene driven by the *rolD* promoter was released from plasmid pB172 by digestion with *Hin*dIII and *Xba*I. This cassette was then ligated into pORE-O3 digested with the same restriction enzymes. The pORE-O3 plasmids were introduced separately by electroporation into *A. tumefaciens* strain GV3101 with helper plasmid pMP90 (Koncz and Schell, 1986) and selected on LB agar medium containing 50 μg rifampicin ml^-1^ and 50 μg kanamycin ml^-1^. *A. tumefaciens* GV3101 was grown in LB medium and used to transform *C. sativa* DH55 using the floral dip method (Liu et al., 2012). Transformed plants were selected by applying phosphinothricin (glufosinate) to 4 day-old seedlings. Integration of the *acdS* cassette into the camelina genome was confirmed by PCR amplification of a 1017 bp region with the following primer set; 5′-ATGAACCTGAATCGTTTTGA-3′ (forward) and 5′-TCAGCCGTTGCGAAACAGGA-3′ (reverse).

The number of T-DNA insertion events was determined using droplet digital PCR (Whale et al., 2012) as follows. The phosphinothricin acetyltransferase (*pat*) selectable marker gene was amplified using the primer set 5′-ACAGAGCCACAAACACCAC-3′ (forward) and 5′-GCAATACCAGCCACAACAC-3′ (reverse) and detected with the 6-carboxy-fluorescein (FAM)-labeled probe 5′-CTCAACCTCAGCAACCAACCAAGGGTA-3′. The *Actin2* reference gene was amplified using the primer set 5′-GCTCTTCCATCGAGAAGAACTAC-3′ (forward) and 5′-CAAACGAGGGCTGGAATAAGA-3′ (reverse) and detected with the hexachloro-fluorescein (HEX)-labeled probe 5′-TGGGCATCTGAATCTCTCAGCACC -3′. DNA was isolated from leaves using the Bio Sprint DNA plant kit (Qiagen) and quantified with a Qubit 2.0 fluorometer (Invitrogen). Ten ng of DNA digested with *Xba*I was added to the PCR reaction mixture with 2 X Supermix (Bio-Rad), 10 μM of each primer and 3.25 nM of each probe to form a final volume of 20 μl. Each reaction was loaded into a DG8 cartridge (Bio-Rad) with 70 μl of droplet generation oil and then transferred into a 96-well PCR plate. The plates were heat-sealed and then cycled under the following conditions: 95°C for 10 min (one cycle), 40 cycles of 94°C for 30 s and 56°C for 1 min, followed by 10 min of 98°C. After PCR, the plates were placed on a QX200 droplet reader (Bio-Rad) to quantify positive droplets. Data were analyzed using Quanta-Soft version 1.7.4.0917 (Bio-Rad) and insert copy number calculated relative to the *Actin2* reference gene which is present in three copies in the allohexaploid camelina genome.

Copy number variation (CNV) was calculated based on the following formula, CNV = (A/B)NB, where A represents the concentration of target species, B represents the concentration of reference species and N represents the number of copies of reference loci in the genome (Bio-Rad droplet digital PCR application guide, Whale et al., 2012). Individual single copy lines were allowed to self-pollinate and two independent homozygous lines with *acdS* under the control of the CaMV *35S* promoter (lines 27 and 53) and three independent homozygous lines with *acdS* under the control of the *rolD* promoter were selected for further investigation.

The expression of the *P. putida acdS* in transgenic lines was determined using droplet digital PCR as follows. The *acdS* gene was amplified using the primer set 5′- GGCAAACCGTTTCCAATTCC -3′ (forward) and 5′- GTCAAACTTGAAGCCCAAATCC -3′ (reverse) and detected with the 6-carboxyfluorescein (FAM)-labeled probe 5′- CTCGGGTTTGTCGGCTTCGCTA -3′. The *Actin2* reference gene was amplified using the primer set 5′-GCTCTTCCATCGAGAAGAACTAC-3′ (forward) and 5′-CAAACGAGGGCTGGAATAAGA-3′ (reverse) and detected with the HEX-labeled probe 5′-TGGGCATCTGAATCTCTCAGCACC -3′. RNA was isolated from leaves or roots using the RNeasy Plant Mini Kit (Qiagen). cDNA was synthesized using SuperScript III First-Strand Synthesis kit (Thermo Fisher Scientific). 1 ng of cDNA was added to the PCR reaction mixture with 2X Supermix (Bio-Rad), 10 μM of each primer and 3.25 nM of each probe to form a final volume of 20 μl. Each reaction was loaded into a DG8 cartridge (Bio-Rad) with 70 μl of droplet generation oil and then transferred into a 96-well PCR plate. The plates were heat-sealed and then cycled under the following conditions: 95°C for 10 min (one cycle), 40 cycles of 94°C for 30 s and 56°C for 1 min, followed by 10 min of 98°C. After PCR, the plates were placed on a QX200 droplet reader (Bio-Rad) to quantify positive droplets. Data were analyzed using Quanta-Soft version 1.7.4.0917 (Bio-Rad) and the relative ratio of *acdS* gene expression was calculated relative to the expression of *Actin2* reference gene by plotting the concentration of FAM over the HEX labeled probe according to the Bio-Rad droplet digital PCR application guide.

### Bacterial and Salt Treatments

Bacteria were cultured for 24 h in tryptic soy broth (TSB) containing 100 μg ml^-1^ ampicillin for wild-type strains or 100 μg ml^-1^ ampicillin and 10 μg ml^-1^ tetracycline for the *acdS* mutant strains (Ali et al., 2012). Bacterial cultures were centrifuged at 4,000 × *g* and resuspended to an OD_600__nm_ = 0.50 ± 0.02 in 0.03 M MgSO_4_. Soil was inoculated with either 2 ml of PGPB or 2 ml of 0.03 M MgSO_4_ (control) at the time of sowing and again 1 week after sowing.

A saline solution that closely resembles the composition found in soils on the Canadian Prairies (Steppuhn et al., 2010) was prepared by mixing 1.70 gL^-1^ CaCl_2_, 1.44 gL^-1^ NaCl, 0.80 gL^-1^ MgSO4^.^7H_2_0, 7.90 gL^-1^ Na_2_SO_4_ to obtain a solution with an electrical conductivity (EC) of 15 dSm^-1^, or 2 gL^-1^ CaCl_2_, 1.89 gL^-1^ NaCl, 1.25 gL^-1^ MgSO4^.^7H_2_0, 13.01 gL^-1^ Na_2_SO_4_ to obtain a solution with an EC of 20 dSm^-1^ or 2 gL^-1^ CaCl_2_, 1.87 gL^-1^ NaCl, 1.65 gL^-1^ MgSO4^.^7H_2_0, 21.45 gL^-1^ Na_2_SO_4_ to obtain a solution with an EC of 27 dSm^-1^at 20°C. Camelina seeds were sown in soil-less potting mixture (Stringham, 1971) in 10 cm × 10 cm × 8 cm square pots. All materials are available locally except fritted trace elements (Frit Industries Inc., Ozark, AL, USA). 50 ml of tap water (50 ml at EC 1,248 μSm^-1^) was applied daily to each pot and replaced with 50 ml of saline solution (EC 15 or EC 20 dSm^-1^) 19 days after sowing. The accumulation of salt in the pots was controlled by draining and the EC of the drained water was measured weekly. Plants were grown in a controlled greenhouse environment (16 h light/8 h dark, 20/17°C) supplemented with halogen lights. Growth was measured weekly. Shoot and root matter was harvested 20 days after the initial salt treatment when plants began to flower at which time the length, and fresh and dry weights were recorded. Seeds were collected 60–70 days after sowing when the siliques were completely dried and the seeds were brown.

The weight of 100 seeds and total seed production/plant were measured from 4 to 9 individual replicates. Germination assays were conducted by placing 20 seeds in a 10 cm Petri dish on Whatman #4 paper moistened with either tap water or saline solution (15, 20, or 27 dSm^-1^). The length of the primary roots and hypocotyls was recorded 6 days after germination.

### Extraction and Analysis of Oil, Fatty Acids, and Glucosinolates

Seed oil fatty acyl composition was analyzed using gas chromatography (GC) following preparation of fatty acid methyl esters by base-catalyzed methanolysis (Thies, 1971). Briefly, 0.1 g of seeds of each sample were placed in a 7 mL polycarbonate vial containing a steel rod. 1 mL of tripentadecanoin in hexane at 6 mg/mL was added as an internal standard to each vial before capped vials were put in an Eberbach reciprocating shaker for 20 min (280 rpm, 37 mm stroke). After centrifugation at 2300 rcf for 15 min, 0.05 mL of the supernatants were transferred to 2 mL autosampler vials. After allowing hexane to evaporate naturally in a fume hood for 1 h, hexane (0.05 mL) and 2% sodium methoxide (0.1 mL) were added to each vial. The contents of the vials were mixed and placed at room temperature (20°C) for 15 min. 0.05 ml of 1.7 M sodium phosphate buffer (pH 6.75) was added to each vial. Methanol was evaporated under a stream of air for 2 min. 0.5 mL of heptane was added and 1 μL of each sample was loaded onto an Agilent 6890N GC equipped with a flame ionization detector, an Agilent INNOWAX fused silica capillary column (7.5 m × 250 μm diameter × 0.5 μm film thickness) and split injection (Agilent Technologies Canada Inc., Mississauga, ON, Canada). The injector and detector temperature were 250 and 300°C, respectively. The initial oven temperature was 190°C. After sample injection, oven temperature was increased to 255°C at a rate of 40°C min^-1^ and maintained at 255°C for 1.6 min. The split ratio was 40:1. Data were analyzed using Agilent ChemStation software. Peaks were identified by comparing their retention times with the fatty acid methyl ester standard mixture GLC-428 (Nu-CHEK Prep Inc., Elysian, MN, USA). Individual fatty acids were reported as a percent of total fatty acid methyl esters by mass. Oil contents were calculated by totaling contents of individual triglycerides.

Glucosinolates were extracted, purified and converted to desulfoglucosinolates based on procedures of Thies (1980) and Raney et al. (1995) with modification. Briefly, about 0.1 g of seed was placed in an 8 ml polypropylene vial containing a steel rod. 3 mL of methanol and 1 mL of 1 mM allyl glucosinolate (as an internal standard) were added to each vial before capped vials were put in an Eberbach reciprocating shaker for 60 min (280 rpm, 37 mm stroke). After centrifugation at 2300 rcf for 15 min, 2 ml of the supernatant was loaded onto a mini-column containing 0.3 mL of pre-swollen DEAE-Sephadex anion-exchange resin. The resin was then washed with 1.5 mL 2% acetic acid, 1.8 mL water and 1.2 mL 20 mM sodium acetate at pH 4.0 before adding 0.1 mL of sulfatase to the top of the resin and indubating overnight at room temperature (20°C) for on-column enzymatic desulfation. Desulfoglucosinolates were eluted with 1.2 mL of water and analyzed based on the procedures of American Oilseed Chemists Society Official Method Ce li-07 (American Oil Chemists’ Society, 2009; Pang et al., 2009) with modifications. Briefly, the eluate was filtered through a 0.2 μm pore-size filter. A portion (1–10 μL) of the filtrate was loaded onto a Waters Acquity UPLC system equipped with a Waters Acquity UPLC BEH shield RP18 column (1.7 μm, 2.1 mm × 50 mm) at 30°C using a flow rate of 0.8 mL min^-1^ and a binary solvent system. The solvent gradient was programmed as follows: water (0.3 min), a linear gradient of 0–20% acetonitrile (5.7 min) and 20% acetonitrile (1 min). Analysis was performed with a Waters PDA (photodiode array detector), a TQ (tandem quadrupole) detector and Waters Empower version 3 software. Neutral loss scan (m/z 162.2) was used to confirm the identities of desulphoglucosinolates, while the peak areas at 229 nm normalized against a known amount of added allyl glucosinolate were used for glucosinolate quantification. The UV relative response factor “one” was used for the three camelina desulphoglucosinolates and desulpho allyl glucosinolate.

### Statistical Analysis

Plant growth measurements were expressed as the mean ± standard error for each treatment. Significant differences between treatments were determined by variance analysis (ANOVA) with a *p*-value of ≤0.05 and pair wise comparisons were conducted using the Tukey’s Studentized Range (HSD) test using SAS Software 9.3 (TS1M2).

## Results

### Inoculation of Soil with PGPB

#### Effect of the PGPB on Shoot Length and Weight

**FIGURE 1:**
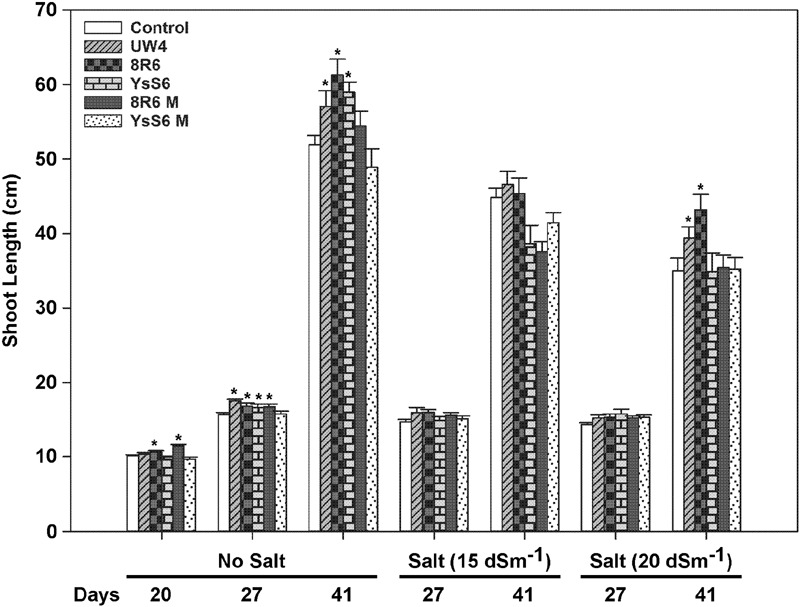
**The effect of soil inoculation with plant growth-promoting bacteria (PGPB) on the shoot length of camelina plants grown in the absence, or presence of, salt (15 and 20 dSm^-1^).** Soil was treated with buffer (control), *Pseudomonas putida* UW4, *P. migulae* 8R6 or its *acdS* mutant 8R6M, *P. fluorescens* YsS6 or its *acdS* mutant YsS6M. Salt was applied 20 days after sowing. Error bars indicate standard error (*n* = 15). A two-way ANOVA and Tukey post-test were used to detect significant differences between groups. Asterisks (^∗^) above bars indicate values that are significantly different (*p* < 0.05) from the control on days when measurements were taken.

Twenty days after sowing, inoculation with UW4 or YsS6 did not have any significant effect on shoot length, but plants grown in soil inoculated with 8R6 or the 8R6M *acdS-* mutant had slightly longer shoots relative to the control (**Figure 1**). 27 days after sowing, plants grown in soil inoculated with all PGPB, except YsS6M, showed significantly increased shoot length in the absence of salt, while 41 days after sowing, plants grown in soil inoculated with any of the three wild-type PGPB had longer shoots under the no salt condition. Soil inoculation with PGPB did not significantly improve shoot length under moderate salt stress (15 dSm^-1^). Under more severe salt stress (20 dSm^-1^), plants grown in soil inoculated with UW4 and 8R6 produced shoots that were 10 and 17 percent longer (*p* < 0.05) relative to the control when the plants started to bolt (41 days after sowing and 21 days after salt treatment), respectively. Contrary to shoot length, plants grown in soil inoculated with UW4 had reduced shoot fresh weight (ca. 20%) in the control and 15 dSm^-1^ salt treatments, while inoculation of soil with the two endophytic PGPB did not affect shoot fresh weight significantly in either the control or salt treatments (data not shown). PGPB did not affect shoot dry weight significantly in either the control or salt treatments (data not shown).

#### Effect of the PGPB on Root Length and Weight

**FIGURE 2:**
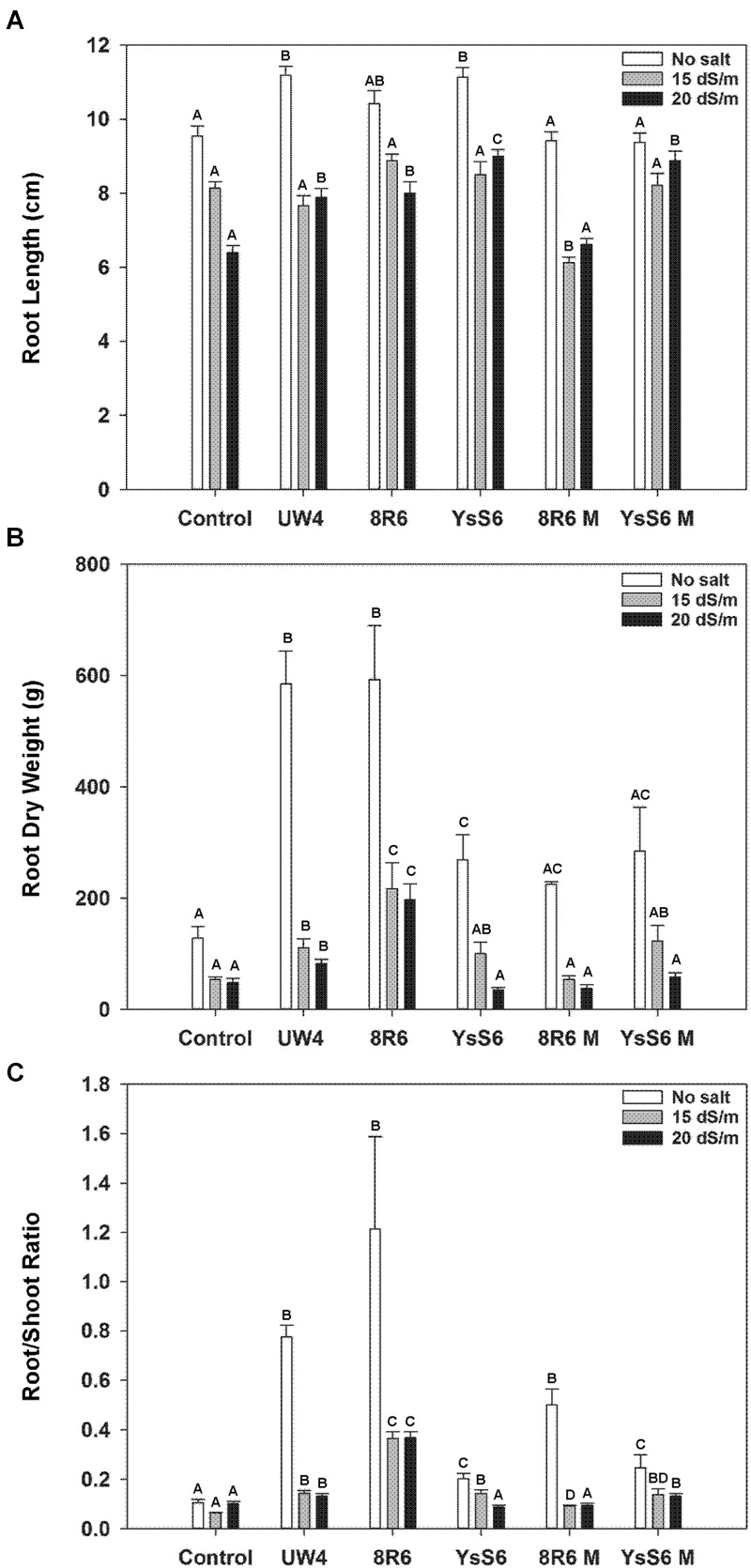
**The effect of soil inoculation with PGPB on root development of camelina plants grown in the absence, or presence of, salt (15 and 20 dSm^-1^).** Soil was treated with buffer (control), *P. putida* UW4, *P. migulae* 8R6 or its *acdS* mutant 8R6M, *P. fluorescens* YsS6 or its *acdS* mutant YsS6M. Salt was applied 20 days after sowing. Panels show root length **(A)**, root dry weight **(B)**, and ratio of root weight to shoot weight 41 days after sowing **(C)**. Error bars indicate standard error (*n* = 15). A two-way ANOVA and Tukey post-test were used to detect significant differences between groups. Capital letters above bars indicate values that are significantly different (*p* < 0.05) between treatments for each salt concentration.

In the absence of salt, inoculation of the soil with any of the wild-type PGPB resulted in plants with significantly longer roots at the time of harvest (41 days after sowing), while inoculation with the *acdS-* mutants had no effect (**Figure 2A**). Root length under moderately saline conditions (15 dSm^-1^) was not affected by inoculation with the wild-type PGPB; however, under more severe salt stress conditions (20 dSm^-1^) plants grown in soil inoculated with PGPB had significantly longer roots than the control. Mutation of the *acdS* gene compromised the impact of both 8R6 and YsS6.

Root dry weight was highly affected by the presence of certain PGPB in both the control and salt treatments (**Figure 2B**). In the absence of salt, root weight was approximately 4-fold greater for plants grown in soil inoculated with UW4 or 8R6, but only 1.5 to 2-fold greater with the other strains. In the presence of salt, root weight was significantly greater for plants grown in soil inoculated with UW4 or 8R6, but not with the YsS6 or the 8R6M or YsS6M *acdS-* mutant strains. In addition, the root dry weight/shoot dry weight ratio was also greater for plants grown in soil inoculated with UW4 or 8R6 in the absence and presence of salt. The same general trend held for YsS6 or the 8R6M or YsS6M *acdS-* mutant strains, but to a lesser degree (**Figure 2C**).

#### Effect of the PGPB on Seed Production

**FIGURE 3:**
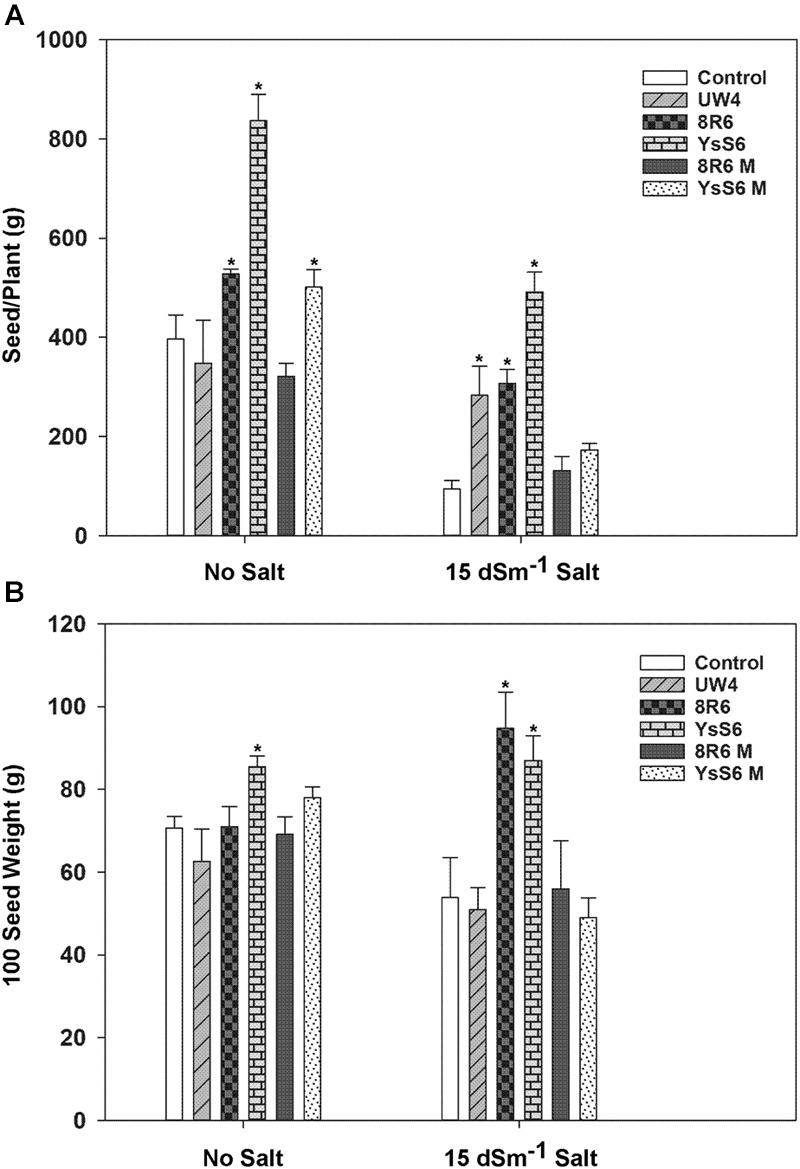
**The effect of soil inoculation with PGPB on seed production of camelina plants grown in the absence, or presence of, salt (15 dSm^-1^). (A)** Shows the amount of seeds produced per plant and **(B)** the weight of 100 seeds. Error bars indicate standard error (*n* = 25 plants in control and 10 plants under moderate salt stress). A two- way ANOVA and Tukey post-test were used to detect significant differences between groups. Asterisks (^∗^) above bars indicate values that are significantly different (*p* < 0.05) from the control.

The effect of PGPB on plant productivity was determined by measuring total seed production, 100 seed weight and the percentage of plants capable of producing seed in the presence and absence of salt. In the absence of salt, total seed production per plant was greater for plants grown in soil inoculated with the endophytic PGPB strains YsS6, YsS6M and 8R6 (**Figure 3A**). However, under moderately saline conditions (15 dSm^-1^), inoculation with any of the wild-type PGPB significantly increased seed production by approximately 30–50% compared to the control, but not with their corresponding *acdS-* mutants. This could not be evaluated under the more severe saline condition (20 dSm^-1^) since most plants did not survive long enough to set seed. In the absence of salt, the quality of the seeds as measured by 100 seed weight was only affected by inoculation with YsS6 (**Figure 3B**), while under moderately saline conditions inoculation with YsS6 and 8R6 yielded seeds with significantly higher 100 seed weight. This effect was not observed in the corresponding *acdS-* mutants.

### Expression of ACC Deaminase in Transgenic Camelina

The studies with PGPB and their corresponding *acdS-* mutants described above indicated that ACC deaminase may be an important, though likely not the only, factor contributing to the beneficial effects of these bacteria on plant growth and development. To explore this aspect further, single insert, homozygous transgenic camelina lines were generated in which the *P. putida* UW4 *acdS* gene was expressed under the control of a broadly constitutive promoter (CaMV *35S*), as well as a root-specific promoter (*rolD*). Droplet digital PCR showed that the two promoters effectively directed the expression of the *acdS* gene in the roots (*35S* and *rolD*) and vegetative tissue (*35S* only) of transgenic camelina plants (**Table 1**). In roots, expression of the *acdS* gene with the *35S* promoter was approximately 11 times higher than with the *rolD* promoter. The expression of the *acdS* gene in vegetative tissues of lines with the *rolD* promoter was not significantly different from the empty vector control indicating that the promoter is highly root-specific.

**Table 1:** Expression of the ACC deaminase (*acdS*) gene in transgenic camelina.

Lines^1^	Shoot	Root
DH55	0 (a)^2^	0.001 ± 0.0009 (a)
35S::27	5.60 ± 1.5 (b)	2.246 ± 0.1 (b)
35S::53	4.42 ± 0.7 (b)	1.171 ± 0.5 (b)
rolD:18	0.002 ± 0.001(a)	0.19 ± 0.07 (c)
rolD:60	0.043 ± 0.026 (ac)	0.12 ± 0.04 (c)
rolD:74	0.010 ± 0.003 (c)	0.12 ± 0.04 (c)
pORE-03	0.001 ± 0.003 (a)	0. 001 ± 0.0004 (a)

*^1^DH55 (wild type control); pORE-03 (empty vector control); lines expressing *acdS* under the control of the CaMV *35S* or *rolD* promoters. ^2^Expression levels are relative to the *Actin2* reference gene of *Camelina sativa* strain DH55. Values represents the mean of three replicates ± SE. In each column, values followed by the same letters in brackets indicate that they are not significantly different (*P* < 0.05).*

#### Germination Assay of Transgenic Camelina

**FIGURE 4:**
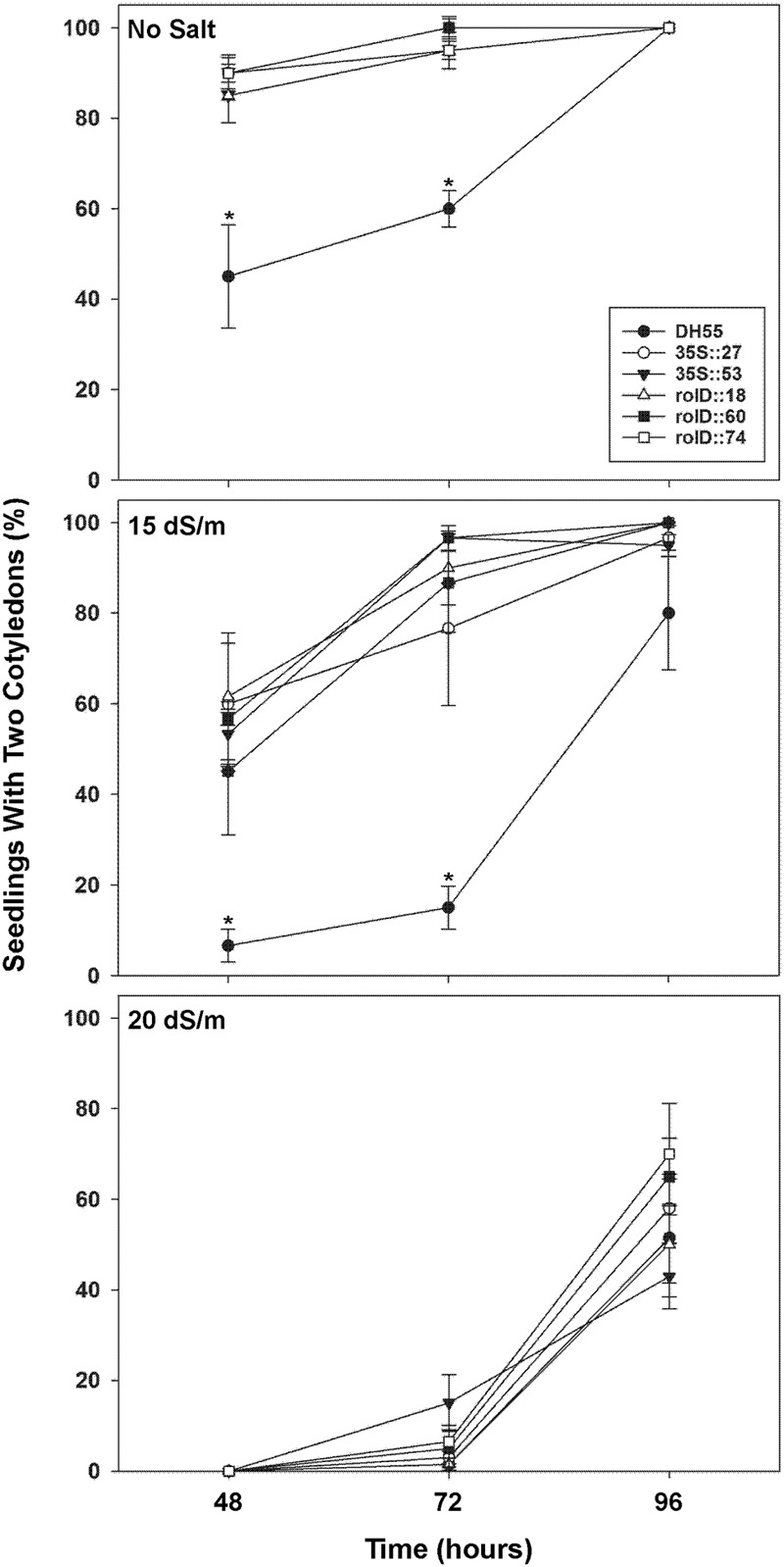
**The effect of *acdS* expression on the rate of germination of camelina seeds in the absence, or presence of, salt (15 and 20 dSm^-1^).** Lines tested include wild-type *C. sativa* DH55 or independent, single insert, homozygous transgenic lines expressing the *acdS* gene under the control of the root-specific *rolD* promoter or the constitutive CaMV *35S* promoter. Error bars indicate standard error [*n* = 60 seeds divided in three plates (20 seeds/ plate)]. A two- way ANOVA and Tukey post-test were used to detect significant differences between groups. Asterisks (^∗^) indicate control (DH55) values that are significantly different (*p* < 0.05) from the transgenic lines.

**FIGURE 5:**
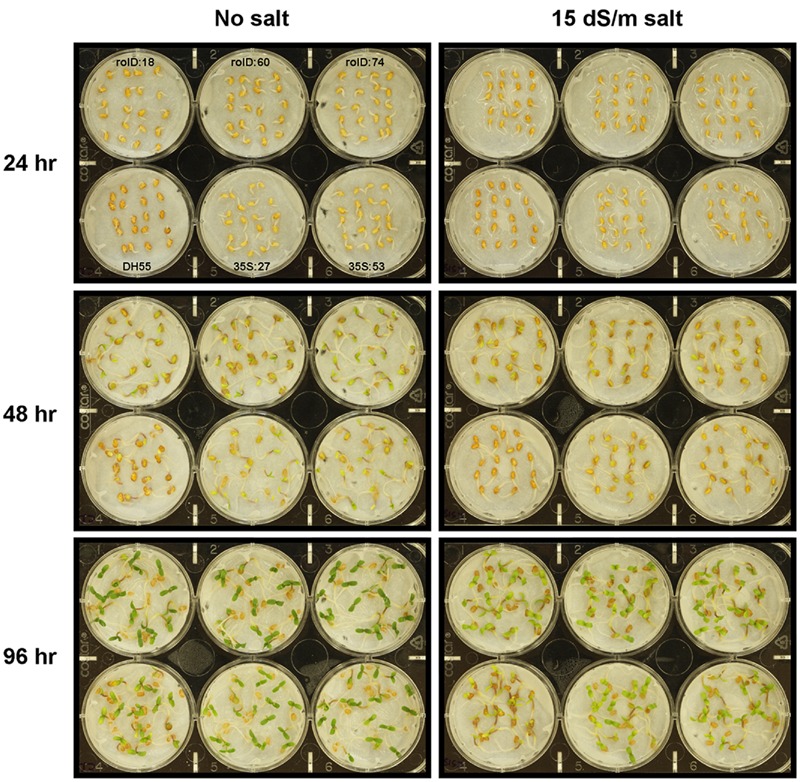
**Germination of camelina seeds in the absence of, or presence of, salt (15 dSm^-1^).** Lines tested include wild-type *C. sativa* DH55 or independent, single insert, homozygous transgenic lines expressing the *acdS* gene under the control of the root-specific *rolD* promoter or the constitutive CaMV *35S* promoter. The grid in the top left panel applies to all panels in the figure.

Radical appearance in seedlings were not affected by *acdS* expression in the absence of, or presence of, moderate salt levels (15 dSm^-1^) during the first 96 h of germination (data not shown). However, the appearance and development of cotyledons were accelerated in all transgenic plants under these conditions compared to the DH55 control (**Figures 4** and **5**). At 48 and 72 h, all of the transgenic lines showed increased seedling development in the absence of salt or under moderate salt levels (15 dSm^-1^). By 96 h, seedling development in the wild type DH55 line reached that of the transgenic lines in that cotyledons emerged from all or almost all of the germinated seeds under these conditions. Under more severe salt stress (20 dSm^-1^), the germination rate was not significantly different between the DH55 and the transgenic lines (**Figure 4**), while at extremely high salt levels (27 dSm^-1^) seeds from all of the lines failed to germinate within 96 h.

**FIGURE 6:**
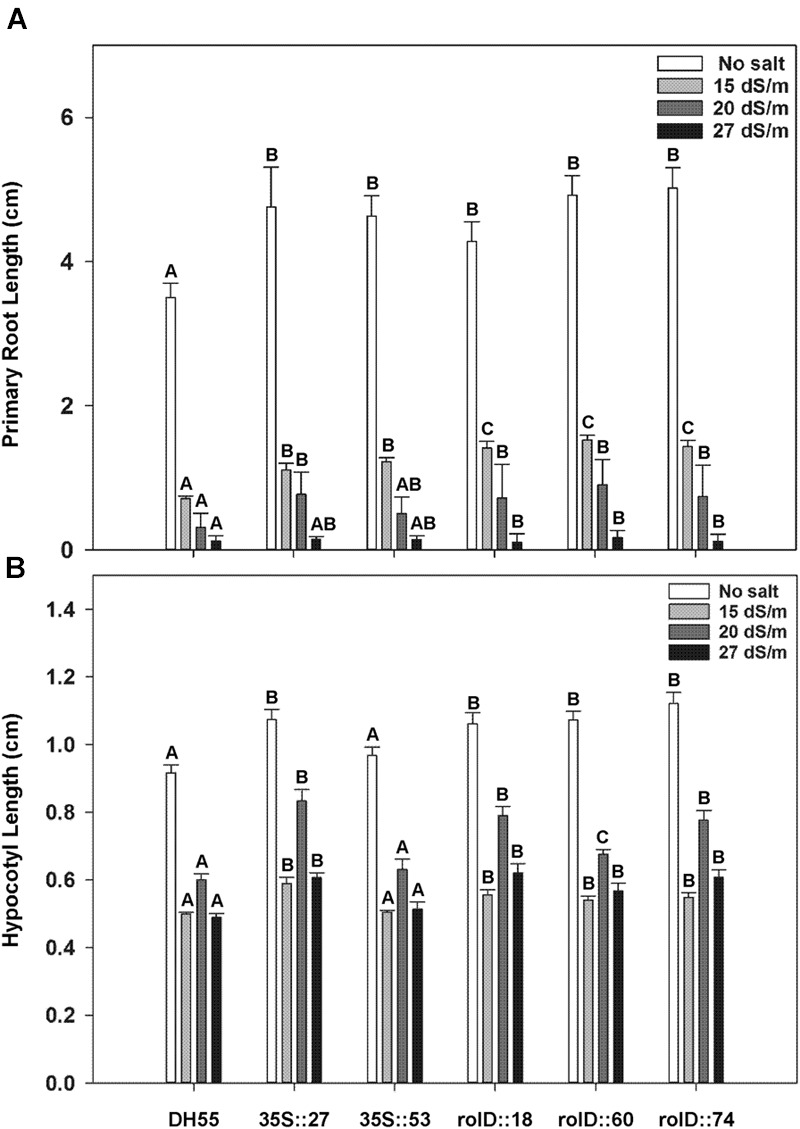
**The effect of *acdS* expression on hypocotyl and primary root length during germination of camelina seeds in the absence, or presence of, salt (15, 20, and 27 dSm^-1^).** Lines tested include wild-type *C. sativa* DH55 or independent, single insert, homozygous transgenic lines expressing the *acdS* gene under the control of the root-specific *rolD* promoter or the constitutive CaMV *35S* promoter. **(A)** Shows primary root length and **(B)** hypocotyl length on day 6. Error bars indicate standard error [60 seeds divided in three plates (20 seeds/plate)]. A two-way ANOVA and Tukey post-test were used to detect significant differences between groups. Capital letters above bars indicate values that are significantly different (*p* < 0.05) between lines for each salt concentration.

When examined 6 days after imbibing water, *acdS* expression also increased the length of the primary root (**Figure 6A**) and hypocotyl (**Figure 6B**) in the absence of salt. While the presence of salt at all levels tested (15, 20, and 27 dSm^-1^) reduced both root and hypocotyl length, the reduction was significantly less in the transgenic lines. Furthermore, lines that expressed the *acdS* gene under the control of the root-specific *rolD* promoter generally had longer roots and hypocotyls than lines using the *35S* promoter grown under salt stress.

**FIGURE 7:**
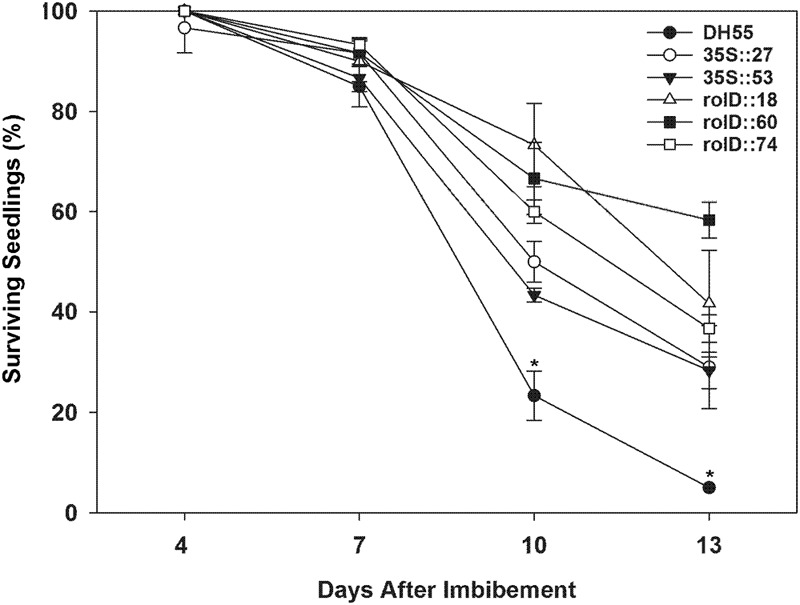
**The effect of *acdS* expression on the seedling survival in the presence of salt (20 dSm^-1^).** Lines tested include wild-type *C. sativa* DH55 or independent, single insert, homozygous transgenic lines expressing the *acdS* gene under the control of the root-specific *rolD* promoter or the constitutive CaMV *35S* promoter. Error bars indicate standard error [60 seeds divided in three plates (20 seeds/plate)]. ANOVA and Tukey post-test were used to detect significant differences between groups. Asterisks(^∗^) indicate control (DH55) values that are significantly different (*p* < 0.05) from the transgenic lines.

**FIGURE 8:**
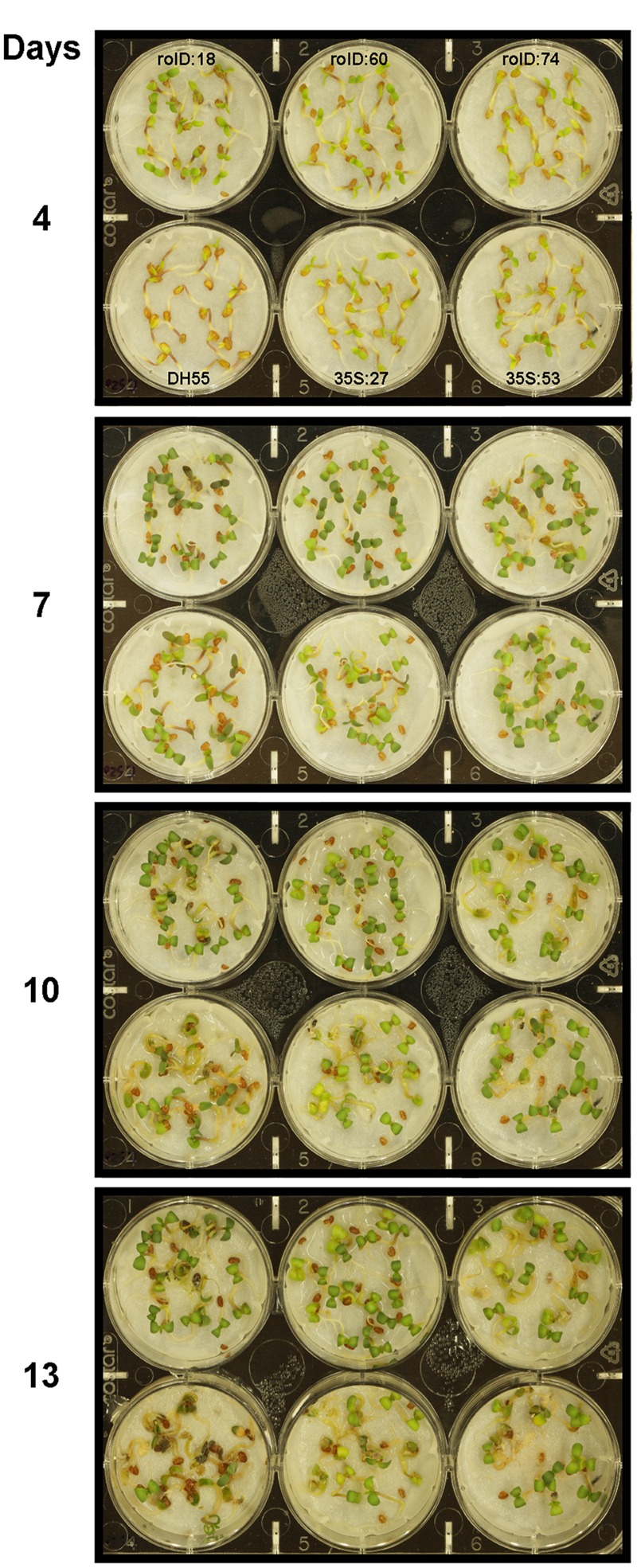
**Growth and survival of camelina seedlings in the presence of salt (20 dSm^-1^).** Lines tested include wild-type *C. sativa* DH55 or independent, single insert, homozygous transgenic lines expressing the *acdS* gene under the control of the root-specific *rolD* promoter or the constitutive CaMV *35S* promoter. The grid in the top panel applies to all panels in the figure.

In the presence of high salt levels (20 dSm^-1^), most of the DH55 seedlings died (senesced) by the 13th day post-imbibition. However, the rate of senescence was reduced in the transgenic lines, especially in lines that expressed the *acdS* gene under the control of the root-specific *rolD* promoter compared to the *35S* promoter (**Figures 7** and **8**).

#### Growth Characterization

**FIGURE 9:**
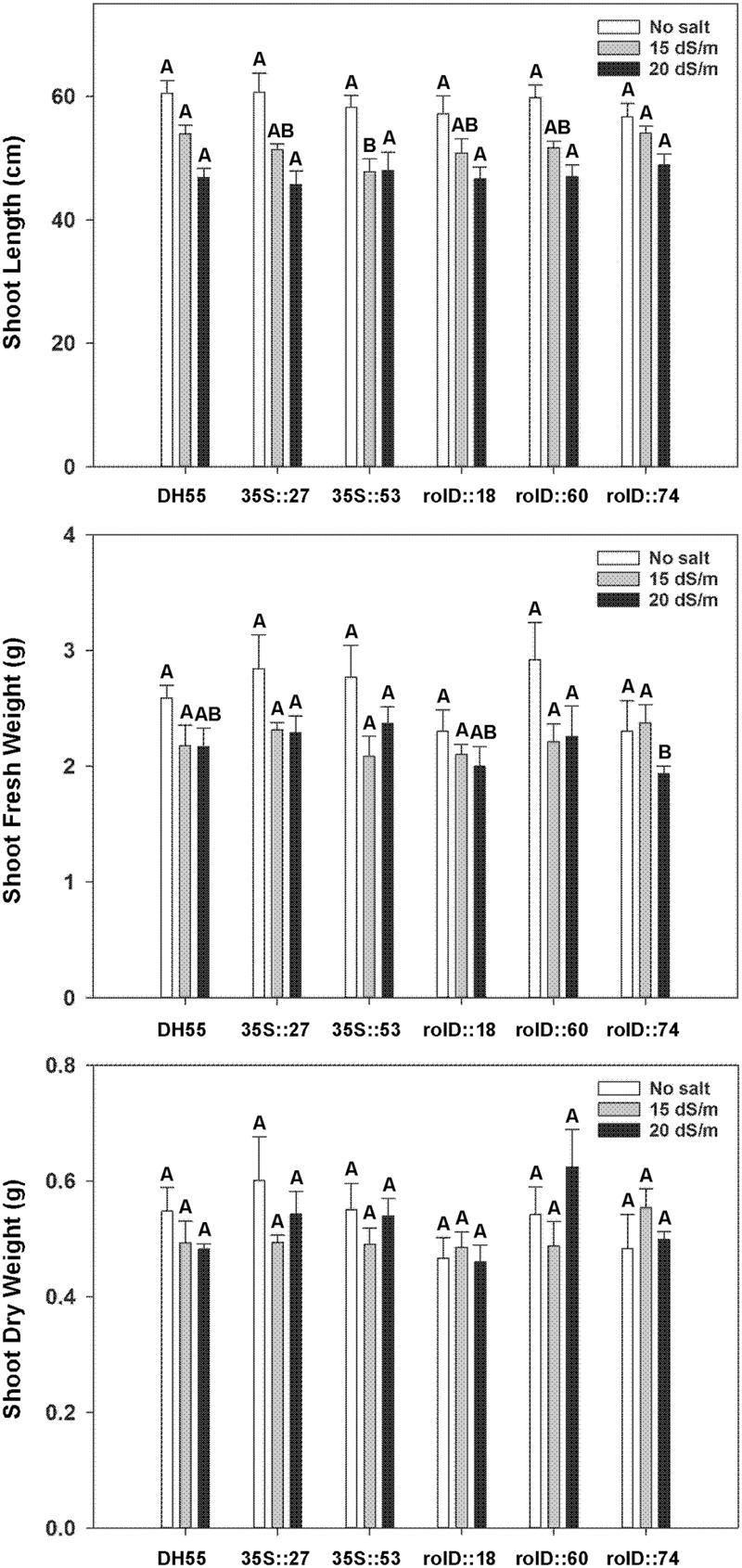
**The effect of *acdS* expression on shoot development of camelina plants grown in the absence, or presence of, salt (15 and 20 dSm^-1^).** Lines tested include wild-type *C. sativa* DH55 or independent, single insert, homozygous transgenic lines expressing the *acdS* gene under the control of the root-specific *rolD* promoter or the constitutive CaMV *35S* promoter. Panels show shoot length, shoot fresh weight, and shoot dry weight 41 days after sowing. Error bars indicate standard error (*n* = 10). A two- way ANOVA and Tukey post-test were used to detect significant differences between groups. Capital letters above bars indicate values that are significantly different (*p* < 0.05) between lines for each salt concentration.

**FIGURE 10:**
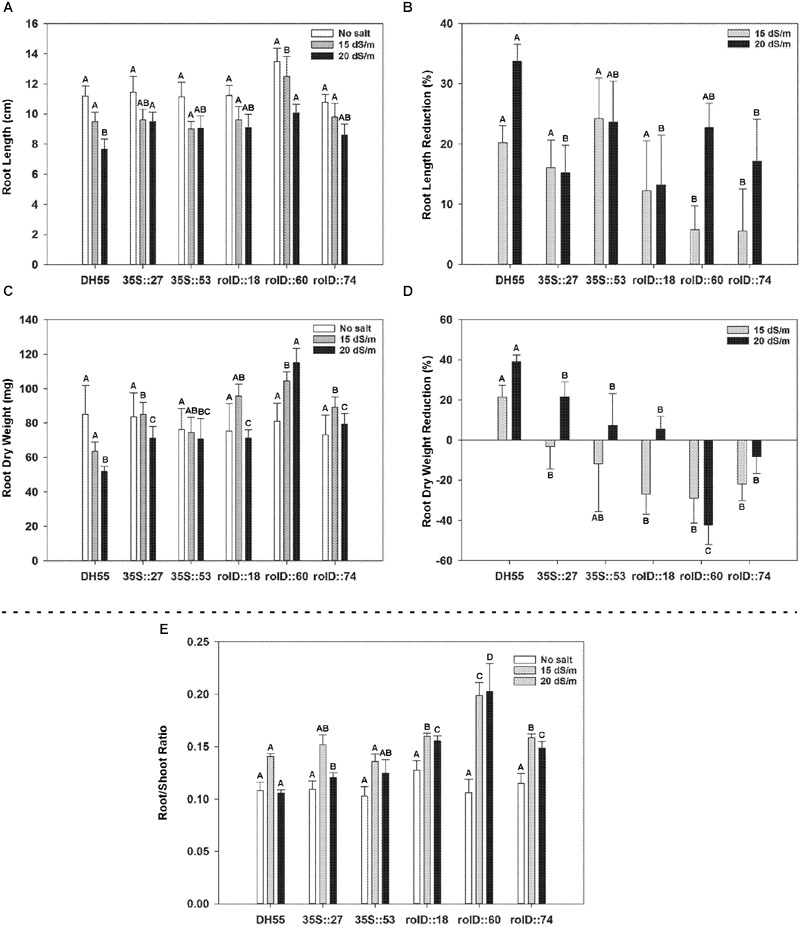
**The effect of *acdS* expression on root development of camelina plants grown in the absence, or presence of, salt (15 and 20 dSm^-1^).** Lines tested include wild-type *C. sativa* DH55 or independent, single insert, homozygous transgenic lines expressing the *acdS* gene under the control of the root-specific *rolD* promoter or the constitutive CaMV *35S* promoter. Panels show root length **(A)**, root length reduction **(B)**, root dry weight **(C)**, root dry weight reduction **(D)**, and ratio of root weight to shoot weight **(E)** 41 days after sowing. Error bars indicate standard error (*n* = 10). A two-way ANOVA and Tukey post-test were used to detect significant differences between groups. Capital letters above bars indicate values that are significantly different (*p* < 0.05) between lines for each salt concentration.

As salt concentration increased, the fresh and dry weights of roots and shoots in both DH55 and the transgenic lines decreased. Shoot fresh and dry weights were not greatly affected by the expression of *acdS* during salt treatments (**Figure 9**); however, the decline in root length and root dry weight during salt treatments was significantly less in the transgenic lines, and again with lines expressing the *acdS* gene under the control of the *rolD* promoter being affected the least (**Figures 10A–D**). As a result, the root/shoot ratio was significantly higher in transgenic lines during salt treatments (**Figure 10E**).

#### Seed Production

**FIGURE 11:**
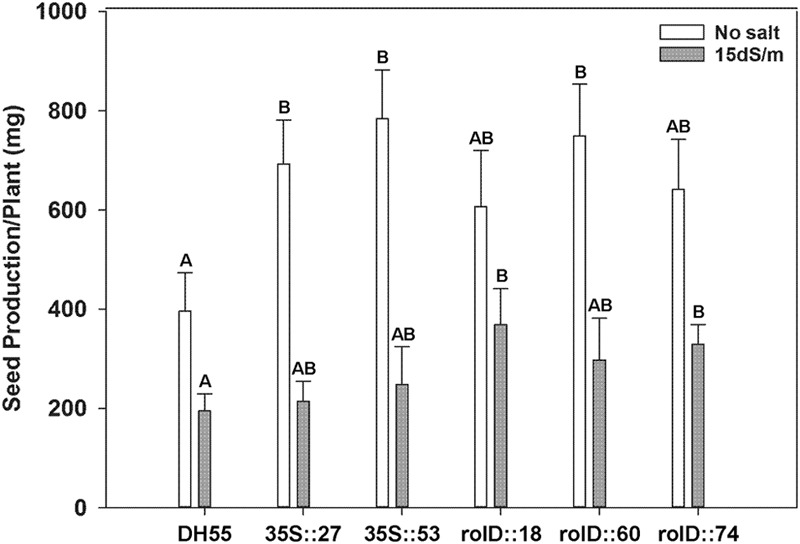
**The effect of *acdS* expression on seed production of camelina plants grown in the absence of, or presence of, salt (15 dSm^-1^).** Lines tested include wild-type *C. sativa* DH55 or independent, single insert, homozygous transgenic lines expressing the *acdS* gene under the control of the root-specific *rolD* promoter or the constitutive CaMV *35S* promoter. Error bars indicate standard error (*n* = 10 plants in control, five plants under moderate and four plants under severe salt stress). A two- way ANOVA and Tukey post-test were used to detect significant differences between groups. Capital letters above bars indicate values that are significantly different (*p* < 0.05) between lines for each salt concentration.

Seed production was substantially higher (1.5-to 2-fold) in all transgenic lines compared to the non-transgenic DH55 line grown in the absence of salt (**Figure 11**). Under the moderate level salt level (15 dSm^-1^), seed yield declined dramatically and only lines expressing *acdS* under the *rolD* promoter showed higher levels of seed production (**Figure 11**). The 100 seed weight was not significantly different between the lines either in the absence, or presence of salt (data not shown). At 20 dSm^-1^, very few plants survived long enough to produce seed and sufficient yield data could not be acquired.

#### Seed Composition

**FIGURE 12:**
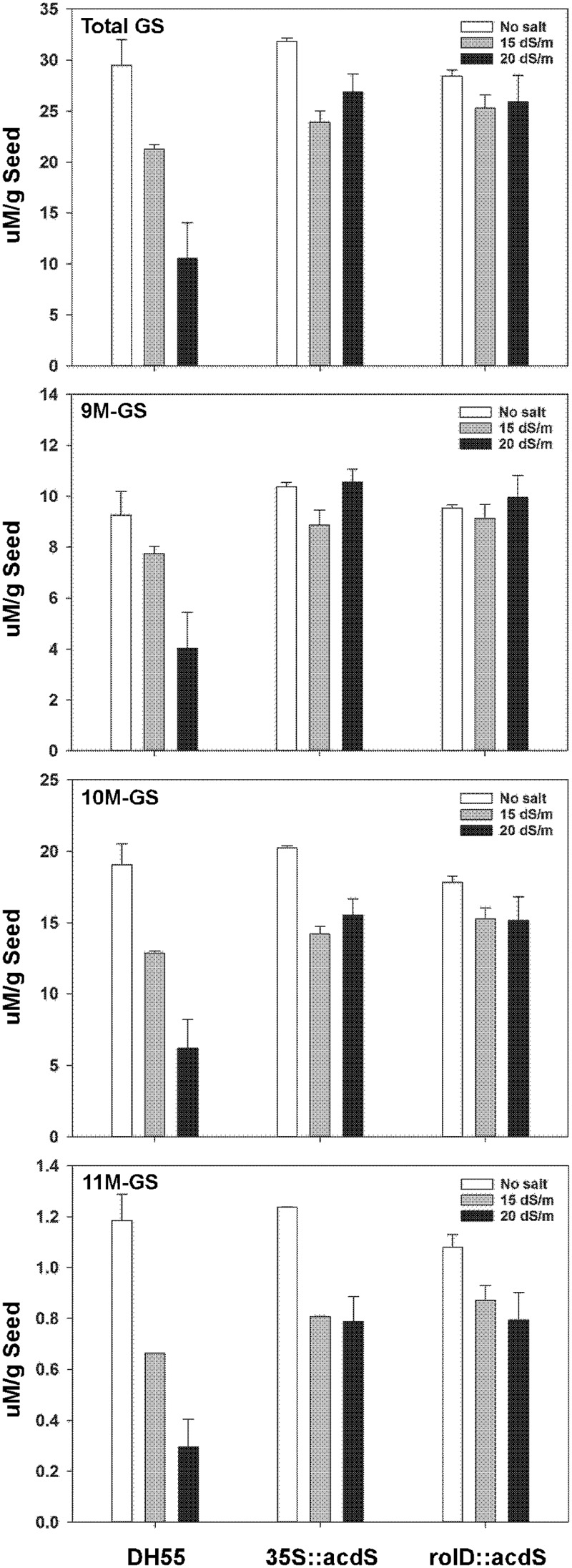
**The effect of *acdS* expression on seed glucosinolate levels in camelina plants grown in the absence of, or presence of, salt (15 and 20 dSm^-1^).** Lines tested include wild-type *C. sativa* DH55 and *transgenic lines expressing the *acdS* gene under the control of theroot-specific *rolD* promoter or the constitutive CaMV *35S* promoter. Error bars indicate the range of two pools of seed each from four plants. Panels show total glucosinolates (GS), 9-(methylsulfinyl)nonyl-glucosinolate (9M-GS), 10-(methylsulfinyl)decyl-glucosinolate (10M-GS), and 11-(methylsulfinyl)undecyl-glucosinolate (11M-GS).*

In the absence of salt, the level of total glucosinolates in the seeds of the wild type DH55 line was similar to that of the transgenic lines, approximately 30 μM g^-1^ seed (**Figure 12**). Three types of aliphatic glucosinolates were detected, namely glucoarabin [9-(methylsulfinyl)nonyl-glucosinolate (9M-GS)], glucocamelinin [10-(methylsulfinyl)decyl-glucosinolate (10M-GS)], and 11-(methylsulfinyl)undecyl-glucosinolate (11M-GS), of which glucocamelinin was the most abundant. In DH55, increasing salt stress resulted in a sharp and corresponding decrease in seed glucosinolate levels. Conversely, seed glucosinolate levels in lines expressing the *acdS* gene under the control of the *rolD* or CaMV *35S* promoters remained the same or decreased only slightly under salt stress.

**FIGURE 13:**
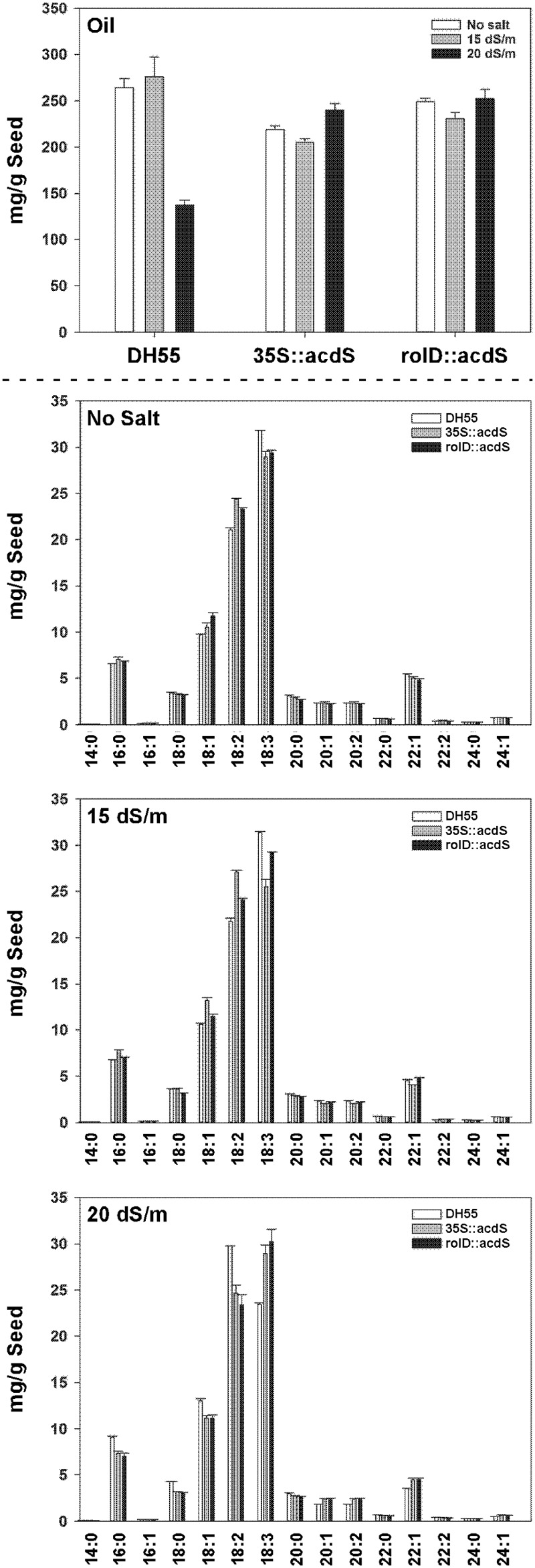
**The effect of *acdS* expression on seed oil and fatty acid content in camelina plants grown in the absence of, or presence of, salt (15 and 20 dSm^-1^).** Lines tested include wild-type *C. sativa* DH55 and *transgenic lines expressing the *acdS* gene under the control of the root-specific *rolD* promoter or the constitutive CaMV *35S* promoter. Error barsindicate the range of two pools of seed each from four plants. Upper panel shows total oil content and the lower panels show fatty acid levelsunder various salt conditions. Fatty acids are as follows: myristic acid (14:0), palmitic acid (16:0), palmitoleic acid (16:1), stearic acid (18:0), oleic acid (18:1), linoleic acid (18:2), α-linolenic acid (18:3), arachidic acid (20:0), 11-eicosenoic acid (20:1), 11,14-eicosadienoic acid (20:2), behenic acid (22:0), erucicacid (22:1), 13,16-docosadienoic acid (22:2), lignoceric acid (24:0), and nervonic acid (24:1).*

The seed oil content of the wild type DH55 line and transgenic lines expressing *acdS* was not affected by the moderate (15 dSm^-1^) salt level (**Figure 13**). Conversely, seed oil content decreased by 50% under severe salt stress (20 dSm^-1^) in the DH55 line, but was unaffected in the transgenic lines under this condition. The fatty acid composition of the DH55 and the transgenic lines followed the same trend as oil content for the main unsaturated fatty acid, α- linolenic acid (18:3), as well as erucic acid (22:1). However, the level of other fatty acids, for example, palmitic (16:0), stearic acid (18:0), oleic acid (18:1), and linoleic (18:2) increased by approximately 25% in the DH55 line under severe salt stress, but remained the same in the transgenic lines.

## Discussion

Camelina is being promoted as an industrial oilseed crop with the potential to occupy marginal lands that are not well-suited for traditional food crops. On the Canadian Prairies, much of this marginal land has a high salt content; however, camelina does not perform any better than canola when both are grown under saline conditions (Steppuhn et al., 2010). Studies of *A. thaliana* mutants affected in salt resistance showed that ethylene signaling regulates plant growth and development in response to salt stress by altering the properties of growth-repressing DELLA proteins (Achard et al., 2006). In addition, the loss of function mutation, ETO1 (Ethylene-Over Production Protein 1), leads to increased ethylene production and higher salinity tolerance by altering Na/K homeostasis (Lockhart, 2013). This suggested that salinity-induced ethylene promotes salt tolerance; however, further enhancement of ethylene levels was found to hamper plant growth and development and results in plants with smaller rosettes, early flowering and reduced seed production (Lockhart, 2013). High ethylene levels also inhibit root elongation and proliferation since treatment with 1-cyclopropenylmethyl butyl ether, an inhibitor of ethylene action in plants, increases canola root length, but has no effect on shoot length (Saleh-Lakha et al., 2004). The effect of high stress ethylene levels in plants can be reduced by plant-associated bacteria that produce ACC deaminase, an enzyme that consumes the biochemical precursor for ethylene. This reduces stress ethylene and rescues normal plant growth while inducing systemic tolerance to stress in plants (Mayak et al., 2004; Glick et al., 2007; Yang et al., 2009; Lim and Kim, 2013; Singh et al., 2015). In the current study, we examined to the effect of inoculating soil with PGPB that produce ACC deaminase, as well as their corresponding *acdS-* mutants, on camelina salt tolerance. The study also evaluated transgenic camelina lines expressing *acdS* under the direction of a strong constitutive promoter (CaMV *35S*) or a root specific promoter (*rolD*).

In the current study, inoculation of soil with both rhizospheric and endophytic PGPB increased shoot length and root development in camelina, which was not observed with bacteria in which the *acdS* gene was disrupted. The effect of PGPB was most profound on root development with a notable increase in the root/shoot ratio in response to salt treatment. This likely influenced the plant reproductive phase, resulting in a significantly higher level of seed production in plants grown in the presence of PGPB and in plants transformed with the *acdS* gene. Moreover, oil and glucosinolate content were unchanged in transgenic plants expressing the *acdS* gene, whereas the levels of these substances were negatively affected in non-transformed control plants under salt stress. These results demonstrate the highly positive impact of PGPB on camelina growth and production under normal and salt stress conditions and the contribution of ACC deaminase to improving tolerance to salt. Promotion of plant growth by PGPB producing ACC deaminase has been reported for several other dicot and monocot plants, such as barley, oats, canola and tomato, especially under stress conditions (Farwell et al., 2007; Ali et al., 2014; Chang et al., 2014). As these bacteria can survive and be effective under salt concentrations up to 24 dSm^-1^ (Chang et al., 2014), they have the potential to greatly increase yield potential and extend cropping into very marginal lands.

Our results indicated that both rhizospheric and endophytic PGPB that produce ACC deaminase improve camelina performance under normal growing conditions. This finding is in contrast to observations with canola and tomato where PGPB had little or no effect on plant growth and development in the absence of stress (Glick et al., 2007). Endophytic PGPB also appeared to be more effective than rhizospheric PGPB in maintaining camelina performance under salt stress in our study, likely since the former establish a more intimate association with the host (Ali et al., 2014). However, we cannot attribute all of the positive effects of PGPB on camelina root development to the reduction of stress ethylene by ACC deaminase. In addition to reducing stress ethylene levels, PGPB may also promote plant growth by synthesizing siderophores or growth phytohormones, such as auxin, gibberellin, and cytokinin (Glick et al., 2007). Since different PGPB may use varied mechanisms to impact plant growth and development, they often exhibit different effects under different conditions (Glick et al., 2007; Nascimento et al., 2012). Rapid establishment of roots, either through elongation of the primary root or by proliferation of lateral roots, can also be induced by PGPB through the synthesis of indole acetic acid (IAA) (Patten and Glick, 2002a,b). This may explain some of the contradictory results between the UW4, 8R6, YsS6 wild type strains and their *acdS-* mutants on growth promotion. For example, IAA production in YsS6 (35.15 μg ml^-1^ units) was similar to 8R6 (29.36 units), whereas siderophore production and phosphorous solubilisation activity was much higher in YsS6 than 8R6. Furthermore, ACC deaminase activity in the wild type YsS6 was 12.5 μmol mg^-1^ h^-1^ and the corresponding YsS6M *acdS-* mutant was 0.11 μmol mg^-1^ h^-1^ compared to 10.9 and 0.03 μmol mg^-1^ h^-^1 in the wild type 8R6 and 8R6M *acdS-* mutant, respectively (Ma et al., 2003; Ali et al., 2014). Since ethylene has a wide range of biological activities and is active at concentrations as low as 0.05 > μl L^-1^ (Abeles et al., 1992), the YsS6M *acdS-* mutant strain may still provide some degree of growth promotion. Such contrasting effects on root growth between PGPB and *acdS-* mutants of PGPB were also observed in a study with *A. thaliana* where inoculation with an *acdS-* mutant strain resulted in seedlings with significantly longer root hairs than inoculation with the wild type strain (Contesto et al., 2008). In *Brassica napus*, ethylene production in plants treated with *P. putida* UW4 was reduced in the entire plant during salt treatments (Cheng et al., 2012) indicating that the resultant effects may be systemic and far-reaching. Regardless of the mechanism, camelina appears to be highly responsive to the presence of PGPB, in particular endophytic types, under both normal and stress conditions.

We expressed the *acdS* gene in camelina based on the observation that, in general, PGPB producing ACC deaminase increased growth and productivity under salt stress conditions better than their corresponding mutant strains. This allowed the effect of ACC deaminase to be isolated from the impact of IAA production, siderophore production, phosphorous solubilisation, bacterial location and others variables between PGPB strains. As expected, *acdS* expression under the CaMV *35S* promoter was higher in shoot tissue than in root tissue and ca. 11 times greater than the expression of the gene under the *rolD* promoter in root tissue. Some plants, such as *A. thaliana*, tomato and poplar, also produce ACC deaminase as a means to regulate ethylene levels during plant development and fruit ripening (McDonnell et al., 2009; Plett et al., 2009); however, downregulation of the *A. thaliana acdS* gene did not affect salt tolerance (Plett et al., 2009). The *C. sativa* DH55 genome (Kagale et al., 2014) contains five genes with a high degree of similarity to the *A. thaliana acdS* gene. Nonetheless, expression of the bacterial *acdS* gene in camelina under the control of the CaMV *35S* and *rolD* promoters increased seedling development and length of the primary root in the absence of and presence of salt. Under salt stress, the root specific *rolD* promoter had a more positive effect than the CaMV *35S* promoter on growth and development. Similarly, a more positive effect on salt and nickel stress tolerance was observed in *B. napus* transformed with *acdS* under the direction of the *rolD* than the *CaMV 35S* promoter (Stearns et al., 2005; Sergeeva et al., 2006). Localization of ACC deaminase activity and reduction of ethylene levels only in the root system where the salt stress is incurred was proposed to be the reason for the more positive effect of the *rolD* promoter compared to the CaMV *35S* promoter (Stearns et al., 2005).

In the current study, we found that seed oil content decreased by ca. 50% in the wild type DH55 line under severe salt stress conditions (20 dSm^-1^), but was unaffected in transgenic lines expressing *acdS*. Biotic and abiotic stresses are known to adversely affect seed quantity as well as seed quality in terms of fatty acid composition, oil stability and oil processing (Bellaloui et al., 2013); high salinity affects both the content and quality (composition) of the oil (Beke and Volkmar, 1995). In vegetable oilseed crops, the level of the main seed fatty acids, namely palmitic, stearic, oleic, and linoleic acid, determine the quantity of oil, while oil quality is equated with high levels of mono- and poly-unsaturated fatty acids, such as oleic, linoleic and linolenic acid, which are more desirable for human consumption than saturated fatty acids (Bergman et al., 2006; Bellaloui et al., 2013). In our study, fatty acid composition changed in response to increasing salt stress, even in the transgenic lines where oil content was maintained. In other studies, salt stress markedly reduced oil, linoleic acid and δ-tocopherol content in sunflower, as well as petroselinic acid in sweet fennel, while increasing linolenic acid, palmitic acid, stearic acid, α- and γ-tocopherol levels in sunflower and palmitic acid in sweet fennel (Noreen and Ashraf, 2010; Bettaieb Rebey et al., 2016). In soybean, low water stress (soil water potential between -150 to -200 kPa) or high temperature (40/33°C, day/night) resulted in higher palmitic and oleic acid levels, but lower stearic acid, linoleic and linolenic acid concentrations (Bellaloui et al., 2013). Drought also reduces the amount of linolenic acid in *B. juncea, B. carinata*, and *C. sativa* seed (Enjalbert et al., 2013). Similarly, salt stress increases oleic acid and decreases linoleic acid levels in sunflower, a phenomenon that was attributed to inhibition of delta-12 oleate desaturase (Di Caterina et al., 2007).

High salt concentration also activates metabolic pathways involved in the synthesis and accumulation of secondary products in plants (Guo et al., 2013; Martínez-Ballesta et al., 2013). Glucosinolates are thioglucosides derived from amino acids of which more than 120 types have been identified; many of these have been found in the Brassicaceae and related species (Chen and Andreasson, 2001; Fahey et al., 2001). They are distributed throughout the plant, including leaves, roots, flowers, fruit, and seeds (Textor and Gershenzon, 2009) where they contribute the hot-pungent flavor and aroma to foods such as mustard, but have also been linked to insect resistance in Brassicaceae crops (reviewed in Hegedus and Erlandson, 2010), as well as protection against cancer and heart disease in humans (Traka and Mithen, 2009). Camelina produces three types of aliphatic glucosinolates, namely glucoarabin [9-(methylsulfinyl)nonyl-glucosinolate (9M-GS)], glucocamelinin [10-(methylsulfinyl)decyl-glucosinolate (10M-GS)], and 11-(methylsulfinyl)undecyl-glucosinolate (11M-GS). The current study showed that in wild type camelina, increasing salt stress resulted in a sharp and corresponding decrease in seed glucosinolate levels, while in lines expressing the bacterial *acdS* gene glucosinolate levels remained the same or decreased only slightly. The change in glucosinolate profile in response to environmental factors has brought forward different theories regarding their potential role(s) in the plant stress response. The most accepted theory is that the glucosinolate-myrosinase system is involved in plant defense responses to biotic stresses (Redovnikovic et al., 2008; Martínez-Ballesta et al., 2013). However, an increase in leaf and seed glucosinolate levels also occurs in *Brassica* species in response to salinity stress (Champolivier and Merrien, 1996; López-Berenguer et al., 2008; del Carmen Martínez-Ballesta et al., 2013). This appears to be dependent on the level of stress as the total glucosinolate level in broccoli leaves were lower at 40 mM NaCl, but higher at 80 mM NaCl compared to the control (López-Berenguer et al., 2008). It was suggested that the increase in glucosinolates was related to the synthesis of osmoprotective compounds that may contribute to water retention (del Carmen Martínez-Ballesta et al., 2013; Martínez-Ballesta et al., 2015). This notion is in accordance with our observation that *acdS* expression maintains glucosinolate levels during salt stress. However, the effect of ethylene and its precursor, ACC, on glucosinolate production is puzzling; for example, treatment of *Arabidopsis* leaves with ACC suppressed *N*-methoxyindole-3-ylmethyl glucosinolate production, but induced production of 4-methoxyindole-3-ylmethyl glucosinolate (Mikkelsen et al., 2003). These contradictory effects might be explained in part by the interaction/crosstalk of the abscisic acid, jasmonic acid, salicylic acid, and ethylene pathways which fine tunes the defense response against biotic and abiotic stresses (Martínez-Ballesta et al., 2013, 2014).

## Conclusion

Camelina is being touted as an alternative oilseed crop, in particular for its purported ability to grow on lands not well-suited for traditional food crops (Jiang et al., 2013); however, it does not perform comparatively well to other oilseeds on saline soils (Steppuhn et al., 2010). This study has shown that PGPB can enhance growth and salt tolerance in camelina and that this was due, in part, to the production of ACC deaminase. Physiologically, this appeared to be due to enhanced root system development that would allow the plant to remain productive under stress conditions. Furthermore, expression of *acdS* in transgenic camelina allowed seed production, oil quantity, and glucosinolate levels to be maintained under salt stress. While myriad applications are envisioned for emerging industrial crops, at present, industrial interest in camelina derives mainly from the use of the oil as a biofuel (Li and Mupondwa, 2014), the meal for inclusion in animal (Ariza et al., 2010) and fish feeds (Hixson et al., 2014) or in textiles (Chen et al., 2016), and glucosinolates as natural insecticides. Our research has indicated that these key outputs can be enhanced, or at the very least maintained, while expanding the land base for camelina production to increasingly marginal lands.

## Author Contributions

ZH (post-doctoral fellow) and MY (technician) conducted the research. DH and MG are the principal investigators. BG is a collaborator working on salinity stress and provided the plant growth promoting bacteria and the *acdS* gene. RZ provided analytical services pertaining to glucosinolates, oil and fatty acids. ZY, MG, BG, RZ, and DH wrote the manuscript.

## Conflict of Interest Statement

The authors declare that the research was conducted in the absence of any commercial or financial relationships that could be construed as a potential conflict of interest.
